# In Vitro and In Vivo Anti-Arthritic Potential of *Caralluma tuberculata* N. E. Brown. and Its Chemical Characterization

**DOI:** 10.3390/molecules27196323

**Published:** 2022-09-26

**Authors:** Nida Iftikhar, Ammara Saleem, Muhammad Furqan Akhtar, Ghulam Abbas, Shahid Shah, Shabana Bibi, Ghulam Md Ashraf, Badrah S. Alghamdi, Turki S. Abujamel

**Affiliations:** 1 Department of Pharmacology, Faculty of Pharmaceutical Sciences, Government College University Faisalabad, Faisalabad 38000, Pakistan; 2 Riphah Institute of Pharmaceutical Sciences, Riphah International University, Lahore Campus, Lahore 54000, Pakistan; 3 Department of Pharmaceutics, Faculty of Pharmaceutical Sciences, Government College University Faisalabad, Faisalabad 38000, Pakistan; 4 Department of Pharmacy Practice, Faculty of Pharmaceutical Sciences, Government College University Faisalabad, Faisalabad 38000, Pakistan; 5 Department of Biosciences, Shifa Tameer-e-Millat University, Islamabad 44000, Pakistan; 6 Yunnan Herbal Laboratory, College of Ecology and Environmental Sciences, Yunnan University, Kunming 650091, China; 7 Pre-Clinical Research Unit, King Fahd Medical Research Center, King Abdulaziz University, Jeddah 21589, Saudi Arabia; 8 Department of Medical Laboratory Sciences, Faculty of Applied Medical Sciences, King Abdulaziz University, Jeddah 21589, Saudi Arabia; 9 Neuroscience Unit, Department of Physiology, Faculty of Medicine, King Abdulaziz University, Jeddah 21589, Saudi Arabia; 10 Vaccines and Immunotherapy Unit, King Fahd Medical Research Center, King Abdulaziz University, Jeddah 21589, Saudi Arabia

**Keywords:** *Caralluma tuberculata*, oxidative stress, high performance liquid chromatography (HPLC) and gas chromatography–mass spectrometry, CFA, NF-Κβ

## Abstract

Present research was planned to assess the in vitro and in vivo anti-arthritic potential of *Caralluma tuberculata* N. E. Brown. methanolic (CTME) and aqueous (CTAQ) extracts. Chemical characterization was done by high-performance liquid chromatography and gas chromatography–mass spectrometry analysis. The Complete Freund’s Adjuvant (CFA) was injected in left hind paw of rat at day 1 and dosing at 150, 300 and 600 mg/kg was started on the 8th day via oral gavage in all groups except normal and disease control rats (which were given distilled water), whereas methotrexate (intraperitoneal; 1 mg/kg/mL) was administered to standard control. The CTME and CTAQ exerted significant (*p* < 0.01–0.0001) in vitro anti-arthritic action. Both extracts notably reduced paw edema, and restored weight loss, immune organs weight, arthritic score, RBCs, ESR, platelet count, rheumatoid factor (RF), C-reactive protein, and WBCs in treated rats. The plant extracts showed significant (*p* < 0.05–0.0001) downregulation of tumor necrosis factor-α, Interleukin-6, -1β, NF-κB, and cyclooxygenase-2, while notably upregulated IL-4, IL-10, I-κBα in contrast to disease control rats. The plant extracts noticeably (*p* < 0.001–0.0001) restored the superoxide dismutase and catalase activities and MDA levels in treated rats. Both extracts exhibited significant anti-arthritic potential. The promising potential was exhibited by both extracts probably due to phenolic, and flavonoids compounds.

## 1. Introduction

Rheumatoid arthritis (RA), also known as rheumatism, is a chronic autoimmune disorder associated with tenacious inflammation, stiffness, pain and damage of synovial joints, which lead to physical disability. Symptoms of RA include fatigue, malaise, fever and pain in multiple joints, and prolonged morning stiffness along with various extra-articular manifestations [1]. The worldwide prevalence of RA is 1%. It can occur at any stage of life, but mostly affects at 40–50 years of age. Women are at higher risk than men [2]. The RA is a multifactorial disease associated with the interaction between genetic and environmental factors such as smoking, infections, pollutants etc. [3].

The exact etiology of the disease is not known yet. There is a discharge of autoantibodies that contribute to the progression of disease apart from articular and systemic inflammation [4]. The innate and humoral immunity along with the production of cytokines, matrix metalloproteinases (MMPs), prostaglandins (PGs), cyclooxygenase (COX) and leukotrienes (LTs) plays a vital role in disease pathogenesis and prognosis [5,6]. Bone destruction occurs due to pannus formation in the synovial membrane, whereas secreted enzymes degrade cartilage [7]. Immune responses are directed Th cells. The Th1 cells are responsible for the activation of macrophages and release of pro-inflammatory cytokines such as tumor necrosis factor-α (TNF-α), interleukin (IL)-1, -6, -1β and -7, thus playing a pivotal role in cellular immunity. Furthermore, Th2 cells are known for humoral immunity as they inhibit macrophages and activate the discharge of anti-inflammatory cytokines such as IL-4, -10 and -13 [8]. The excessive load of reactive oxygen moieties (ROS) contributes to the development and progression of various diseases like diabetes, cancer and RA [9].

There is no distinct treatment for the RA; rather, different treatment strategies are adopted for the symptomatic relief and to delay the progression of disease [10]. Commonly prescribed medicines are non-steroidal anti-inflammatory drugs (NSAIDs), disease-modifying anti-rheumatic drugs (DMARDs), biologics (anti-TNF-α) and glucocorticoids [11]. These therapies are lifelong, expensive and causedsevere adverse effects including ulcer, hypertension, hepatotoxicity, cardiovascular diseases, neurodegenerative diseases etc. Due to these major drawbacks, the modern world is moving towards herbal treatments to combat against RA [12]. 

*Caralluma tuberculata* N. E. Brown. (CT), commonly known in Urdu as Chunga and in English as bittercress, is an ethanomedicinally imperious food plant belong to family Apocynaceae. It is a perennial leafless juicy herb, and its stem is consumed as a vegetable by local inhabitants [13,14]. It is found in Pakistan, India, Africa, Spain, and Saudi Arabia [15]. Traditionally, it is used to cure inflammation, stomach problems, diabetes, rheumatism, hypertension, diabetes, pain, and skin problems [16,17]. Previous studies revealed its anti-fungal, anti-bacterial, anti-hyperglycemic, anti-proliferative, hypolipidemic, anticancer, anti-parasitic and gastro-protective activities [15]. Three flavone glycosides and five pregnane glycosides have been isolated from this plant that exhibited anti-inflammatory, analgesic activity and antiparasitic activity [1,18,19]. Previously, anti-arthritic potential of aqueous methanolic extract of shoot was reported [20]. The project was planned to validate and compare the antiarthritic potential of methanolic and aqueous extracts of *C. tuberculata* (whole plant) using in vitro and in vivo tests that may aid in developing an easily available, cost-effective treatment strategy. Additionally, chemical characterization of it by high-performance liquid chromatography (HPLC) and gas chromatography–mass spectrometry (GC-MS) in order to explore its chemical profile. To now, GC-MS analysis of this plant has not been carried out.

## 2. Materials and Methods

Healthy Wistar rats, weighing 120–170 g, were retained in the Animal House of the GC University, Faisalabad (GCUF) at temperature (25 ± 2 °C), humidity (60 ± 5%), for 12 h dark/light cycles with easy access to water and diet. The animals adapted for 2 weeks prior to the start of the study. The approval for animal study protocol was taken from Institutional Review Board, GCUF, (GCUF/ERC/2221). The guidelines of National Institute of Health which are concerned with the care and handling of rodents were followed.

### 2.1. Collection and Extraction of Plant

The whole plant of CT (6 kg) was collected from Faisalabad in November 2020 and identified by a taxonomist of University of Agriculture, Faisalabad, against Voucher no. 1117-20-04. The plant was washed, dried under shade and processed through coarse grinding. To prepare methanolic (CTME) and aqueous extracts (CTAQ), the plant material was separately soaked in methanol and distilled water (DW) (1:5), extracted by a cold maceration process with daily shaking for up to seven days. Both extracts were filtered and passed through Whatman filter papers. The extraction residues were soaked in relevant solvent again and the process was repeated twice. The filtrates were pooled and concentrated by rotary evaporator (Model: RE300, Stuart^®^, Chelmsford, UK) at 40 °C for CTME and 50 °C for CTAQ under reduced pressure. It was further dried to a solid or semisolid mass through air-drying and stored in fridge at 8 °C till further use [9].

### 2.2. Phytochemical Analysis

Phytochemical analysis was carried out using standard procedures described earlier for both extracts for the recognition of secondary metabolites like tannins, phenols, glycosides, flavonoids, saponins, proteins, carbohydrates and alkaloids [9].

### 2.3. HPLC Analysis of CTME

HPLC analysis was executed by adopting an earlier method [21]. The sample was solubilized in a water–methanol (2/3 ratio) solution. A small quantity of HCl was added for acidification and then heated for 2 h at 90 °C. The sample was cooled and filtered with syringe filters (0.2 µm). The filtered sample (20 µL) was inserted to an HPLC (Shimadzu^®^,Kyoto, Japan) fitted with a UV-visible spectrophotometer. The absorbance was measured at 280 nm and then phenols and flavonoids were quantified by comparing retention time of the standards [22].

### 2.4. GC-MS Analysis of CTME

Analysis was carried out using GC-MS (Agilent technologies USA, Santa Clara, CA, USA, GC model: 7890A, MS model: 5977A). The column DB 5MS as stationary phase and 99.9% Helium was set to the rate of 1 mL/min. The oven temperature was set to 50 °C for 1 min then gradually raised at 25 °C/min to 120 °C for 5 min. The inlet was set to 275 °C and sample solution (1 µL) injected into the injector. The MS set to 230 °C for 51.133 min. The standard chromatogram was used to identify unknown compounds [23]. Moreover, all compounds were identified from the retention time of those compounds already detected in GC chromatogram of different plant extracts.

### 2.5. Inhibition of Protein Denaturation 

The egg–albumin–protein denaturation assay was performed by following the earlier method [9]. Different concentrations of test extracts were prepared by double dilution method. The 5 mL of reaction mixture contained egg albumin (0.2 mL), 2.8 mL phosphate-buffered saline (PBS) and 2 mL of plant extracts at different concentrations. The DDW of same volume served as control, while piroxicam as standard control. These were incubated for 15 min, heated at 70 °C and absorbance of reaction mixtures were taken at 660 nm.

The bovine serum albumin (BSA) denaturation procedure described earlier [9] was followed. The test control (0.5 mL) contained 0.45 mL BSA solution and 0.05 mL of DW. The product control comprised of 0.45 mL of double DW and 0.05 mL of sample at various concentrations. The test solution consisted of BSA and test extract at concentrations similar to the product control, while the test standard solution consisted of BSA and standard drug (piroxicam) at similar concentrations as that of extracts. The 1 N HCl was added to adjust the pH (6.3) of all the solutions. The samples were incubated for 20 min and heated for 3 min at 60 °C. The absorbance measured at 660 nm. 

### 2.6. Human Red Blood Cells (HRBC) Membrane Stabilization Assay

The HRBC membrane stabilization assay was performed by the procedure described earlier [9]. Briefly, hypotonic saline, isotonic saline and buffer saline (pH 7.4) were prepared using the standard procedure described earlier. The 3 mL blood was collected from a person, mixed with Alsevier’s solution and centrifuged for 15 min. The separated packed cells were treated with an isosaline solution three times and a 10% *v*/*v* suspension was made with isosaline solution. The test solution comprised of PBS (1 mL; pH 7.2), 2 mL hypotonic saline (0.36% *w*/*v* NaCl), 0.5 mL extract (50, 100, 200, 400, 800 and 1600) and 0.5 mL blood suspension. The solution test control consisted of 2 mL DDW, while the standard solution was comprised of piroxicam in place of the extract solution. The solutions were placed at 37 °C for 30 min and centrifuged for 15 min. The absorbance of supernatant was taken at 560 nm. 

### 2.7. Complete Freund’s Adjuvant (CFA)-Induced Arthritis

The male rats were indiscriminately distributed to nine groups (*n* = 6). The group I served as a normal control (NCG) (healthy rats); group II was the disease control (DCG) (negative control) and were given DW (1 mL). Group III was given standard drugs (methotrexate; intraperitoneal 1 mg/kg once a week); other groups were separately given CTME and CTAQ at 150, 300 and 600 mg/kg for 21 days [24]. Arthritis was induced by inoculating CFA emulsion (0.1 mL) to left hind paw at 1st day to all rats excluding NCG. On day 8 of injecting CFA, treatment was started orally until day 28th.

### 2.8. Evaluation of Arthritis

Before inducing arthritis, initial body weight and paw diameter were measured via digital weighing balance and Vernier caliper respectively before the first immunization (i.e., at day 1) and were further determined on day 7, 12, 16, 20, 24 and 28. The arthritic scoring was also visually recorded and ranged from 0–4 (redness, edema and inflammation) at various days during the course of the study period. 

### 2.9. Evaluation of Arthritis from Blood Parameters

On the 29th day, blood samples were collected in plain and ethylenediamine tetraacetic acid (EDTA) tubes and subjected to hematologic and biochemical testing parameters through the chemical analyzer by using commercially available kits.

### 2.10. Immune Organ Weight and Histopathological Evaluation

All the rats were sacrificed after the 28th day following anesthesia with ether. The spleen and thymus were removed from each rat, clean and weighed. The left hind limb was removed from the rats for histopathological examination [25]. The ankle joints were detached, washed with distilled water and retained in 10% formalin then decalcified by putting them in decalcifying solution for 1 month. The tissues were stained with hematoxylin and eosin (H & E). The ankle joints were observed for inflammation, bone erosion and pannus formation under microscope [26].

### 2.11. Assessment of Oxidative Stress Biomarkers

For this purpose, 10% *w*/*v* liver homogenate was prepared by adopting previous methods [27]. Protein content in tissue was estimated using Lowry’s method as described previously using BSA as standard; absorbance was taken at 660 nm [28].

To estimate superoxide dismutase (SOD) activity, pyrogallol method was adopted [22]. To measure catalase activity, 1.95 mL PBS (pH-7), 0.05 mL of liver homogenate, and 1 mL hydrogen peroxide (30 mM) were taken in a tube and mixed. Absorbance was immediately taken at 240 nm [29]. For MDA levels, thiobarbituric acid method was used [30].

### 2.12. Quantification of Inflammatory Cytokines by Real-time PCR

The pro- and anti-inflammatory cytokines (IL-4, 6, 10, 1β, TNF-α, I-κB, NF-κB, COX-2) were quantified in blood after 28 days of CFA-induced arthritic study by qRT-PCR. For this purpose, RNA extraction was performed from blood by the TRIzol method using a kit from Invitrogen^®^, Waltham, MA, USA followed by synthesis of complementary DNA by adopting Thermo Scientific^TM^, Waltham, MA, USA kit method and kept at −20 °C.

For quantification and amplification, 2X Syber green qPCR master mix kit (GeneAll^®^, Seoul, Korea) method was used to run qRT-PCR (BioRad^®^, System, Hercules, CA, USA). Briefly, a reaction mixture (20 µL) having 10 µL qPCR master mix, 1 µL cDNA, 1.5 µL each of forward and reverse primer and 6 µL of nuclease-free water was prepared and vortexed. The microplates were sealed with transparent film and given a short spin to avoid air bubbles. The programming of qPCR was set for 40 cycles at 90 °C for 2 min, 60 °C for 15 s and at 72 °C for 1 min. The relative expression (2^−ΔΔCT^) of biomarkers were calculated [31]. GAPDH was used as housekeeping gene and primers sequence of cytokines were taken from previous paper [24].

### 2.13. Statistical Analysis

All the data was expressed as mean ± standard deviation (S.D). The data of in vitro assays, paw diameter and body weight were analyzed by two-way analysis of variance (ANOVA) and that remaining by one-way ANOVA followed by Tukey’s test. The level of significance was *p* < 0.05.

## 3. Results

The yields of CTME and CTAQ were 10.78% and 13.1% respectively. The phytochemical analysis indicated the presence of carbohydrates, flavonoids, phenols, tannins and alkaloids in CTME and CTAQ, whereas glycosides, steroids and terpenes were detected in CTME only. Saponins were detected in CTAQ only and proteins were not detected in any extract. 

### 3.1. HPLC Analysis

**Figure 1:**
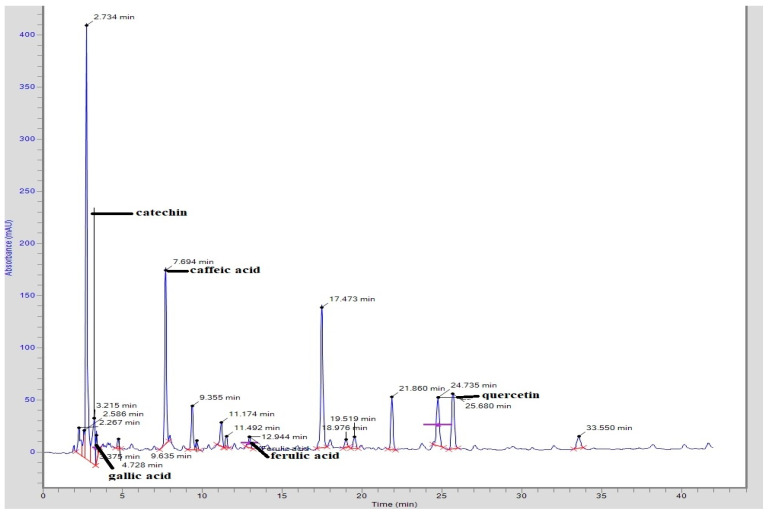
HPLC chromatogram of *Caralluma tuberculata* methanolic extract.

Quantitative estimation of phytochemicals in CTME through HPLC showed that catechin, gallic acid, caffeic acid and ferulic acid were detected in it as mentioned in Table 1 and Figure 1. 

### 3.2. GC-MS

**Figure 2:**
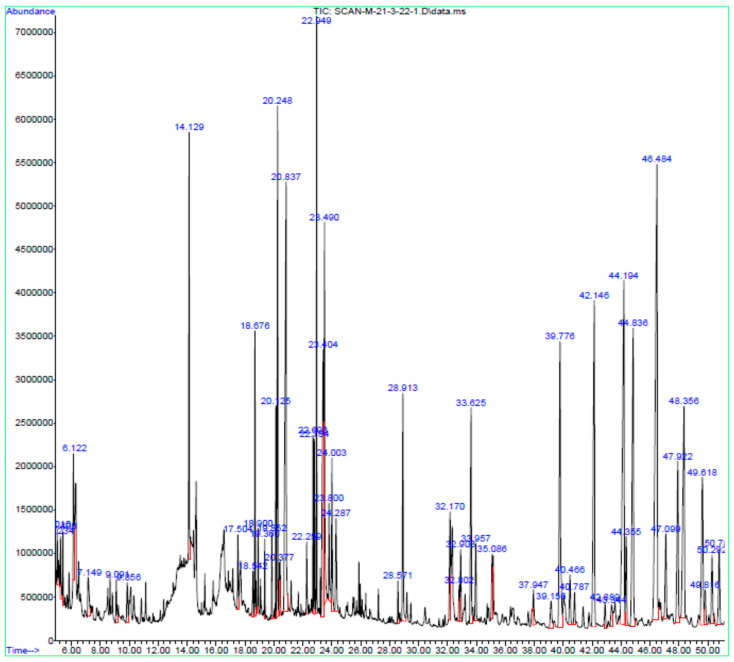
GC-MS chromatogram of *C. tuberculata* extract.

The GC-MS chromatogram of CTME confirmed the presence of various compounds as listed in Table 2 and Figure 2. Major compounds detected in analysis were Hexadecanoic acid, 4-Methyl-2,5-dimethoxybenzaldehyde, Phytol, 9,12-Octadecadienoic acid, 9,17-Octadecadienal, γ-Tocopherol, Vitamin E, Campesterol, Stigmasterol, β-Sitosterol, and Lupeol.

**Figure 3:**
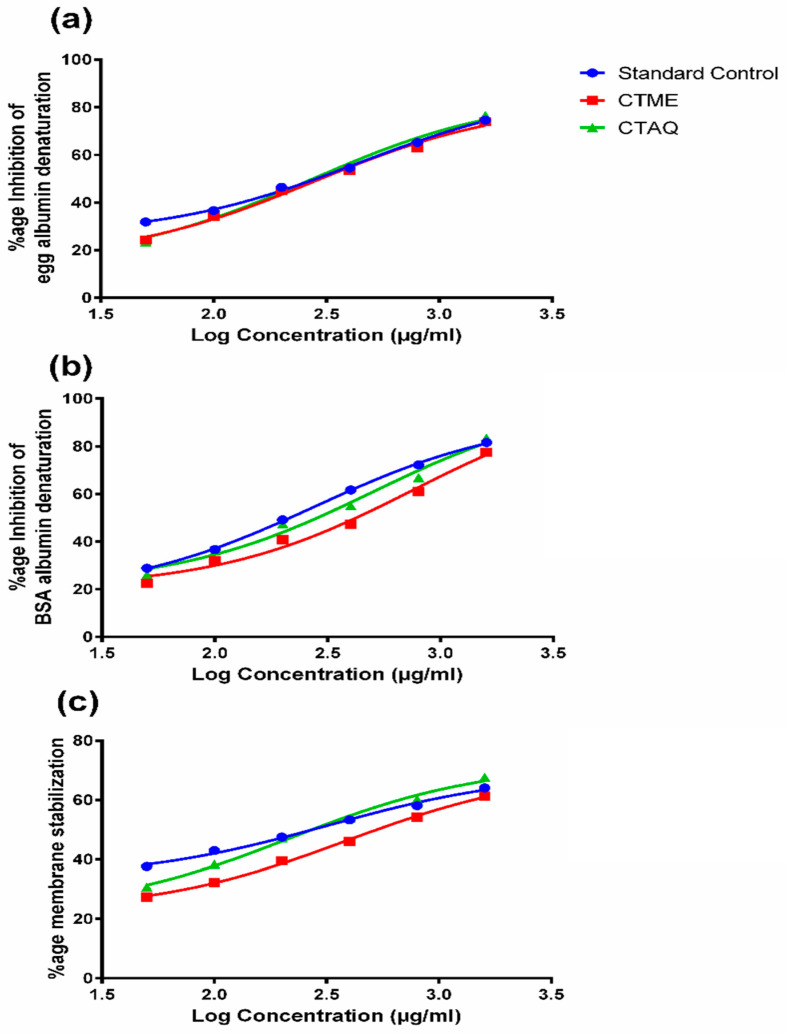
*Caralluma tuberculata* extracts on inhibition of protein denaturation and membrane stabilization. (**a**): Egg albumin denaturation assay; (**b**): BSA denaturation assay; (**c**): HRBC membrane stabilization assay.

Both extracts and piroxicam showed a concentration-reliant rise in percentage inhibition of egg albumin. The CTAQ exhibited highest percentage inhibition (76.53 ± 0.19%) as compared to CTME (73.96 ± 0.03%) and standard (74.62 ± 0.07%) at 1600 µg/mL. Both extracts considerably (*p* < 0.01–0.0001) inhibited protein denaturation as equated to standard, except CTME 800 and CTAQ 400 µg/mL, which were non-significant to the similar concentrations of standard control as shown in Figure 3a. The descending order of IC_50_ (µg/mL) is given as piroxicam (437.6) > CTME (278.2) > CTAQ (271.5).

The anti-denaturation assay using BSA revealed that both extracts inhibited BSA denaturation concentration dependently. The CTAQ showed notably (*p* < 0.001) maximum percentage inhibition (83.41 ± 0.49%) at 1600 µg/mL in contrast to the standard control (81.59 ± 0.55%) and CTME (77.52 ± 0.38) as shown in Figure 3b. Both extracts noticeably (*p* < 0.001–0.0001) inhibited BSA denaturation as compared to the standard except for CTAQ 100 µg/mL that was non-significant at similar concentration of standard control. Decreasing order of IC_50_ (µg/mL) is given as: CTME (753.4) > CTAQ (502.1) > piroxicam (279.4).

### 3.3. Membrane Stabilization Activity

Both extracts exhibited profound (*p* < 0.0001) membrane stabilization dose-dependently and highest percentage protection was noticed with 1600 µg/mL. Whereas, the highest percentage protection was observed with CTAQ (67.64 ± 0.57%) at 1600 µg/mL as compared to standard drug (64.08 ± 0.53%) and CTME (61.32 ± 0.31%) as presented in Figure 3c. Decreasing order of IC_50_ (µg/mL) is given as: CTME (386) > piroxicam (321.3) > CTAQ (217.6).

### 3.4. Effect on Paw Edema

**Figure 4:**
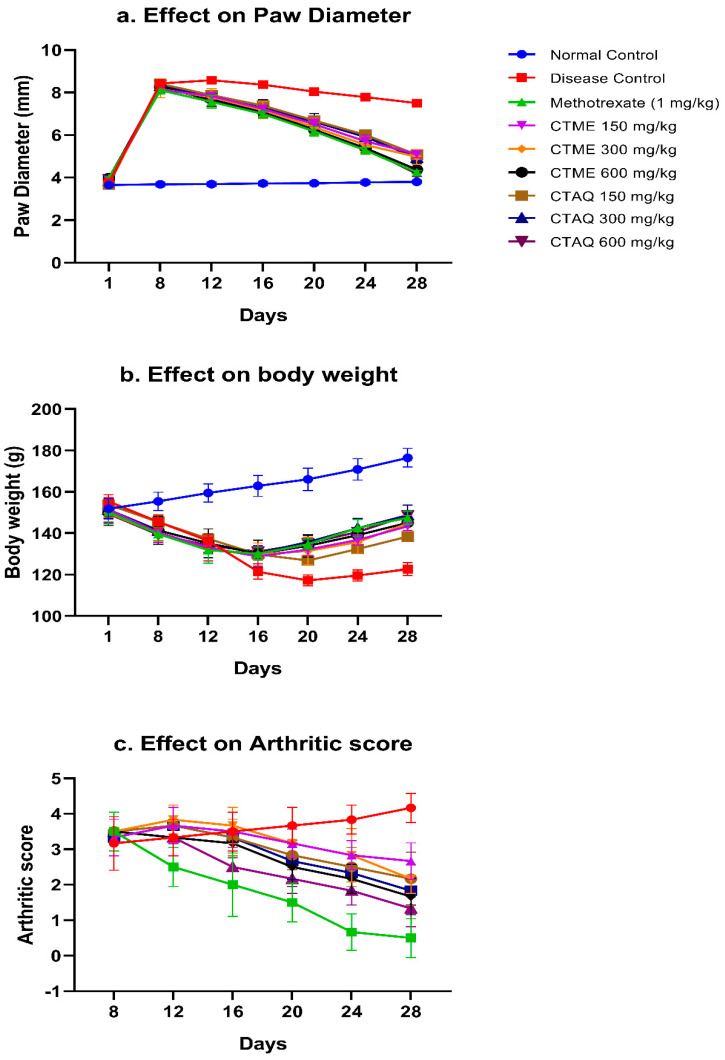
Effect of *C. tuberculata* on the paw diameter, body weight (g) and arthritic index in arthritic rat values as mean ± S.D (*n* = 6). Results were significant *p* < 0.05–0.0001 as equated to DCG.

The CFA administration resulted in inflammation in the injected paw in all rats and prominent swelling was saw at 8th day in all rats as compared to NCG. A noteworthy (*p* < 0.0001) rise in paw diameter was seen in the disease group at 8th day up to 28th day in comparison to normal control, and peak swelling was observed on 12th day. Treatment with both extracts prominently (*p* < 0.0001) decreased paw diameter in contrast to the DCG. Both extracts inhibited paw inflammation dose-dependently and significant percentage inhibition was observed at 600 mg/kg dosage. The maximum inhibitory effect on paw edema was noticed with CTAQ 600 mg/kg (4.18 ± 0.13 mm) on 28th day compared CTME (4.45 ± 0.13 mm) and the standard control group (4.26 ± 0.07 mm) as shown in Figure 4a. A dose-dependent rise in percentage inhibition was observed with both extracts. The maximum percentage inhibition (44.3%) was observed on the 28th day by CTAQ 600 (Figure 4a). The CTME 600 and CTAQ 600 mg/kg non-significantly varied from standard on 28th day.

### 3.5. Body Weight (BW)

There was decreased BW in arthritic rats as disease progressed in contrast to normal rats (Figure 4b). Animals of the disease group indicated a significant (*p* < 0.0001) decrease in body weight from day 8th until the end of study in comparison to normal rats. Whereas CTME, CTAQ (150–600 mg/kg) and methotrexate conspicuously (*p* < 0.05–0.0001) restored body weight as equated to DCG from 16th to 28th day. The loss of body weight was significantly ameliorated by CTAQ 300 (148.67 ± 4.93 g) followed in orders of effects by CTAQ 600 (148.5 ± 1.29 g), standard control (147.4 ± 3.21 g), and CTME 600 mg/kg (145 ± 1.83 g) in contrast to DCG (122.6 ± 3.05 g) at 28th day. The restoration of BW in CTME 600 and CTAQ 600 mg/kg-treated arthritic rats was non-significant as compared to standard control group on 28th day.

### 3.6. Effect on Arthritic Score

A continuous increase in arthritic scoring was exhibited by DCG after CFA inoculation, in contrast to normal rats as shown in Figure 4c. Treatment with methotrexate and plant extracts effectively masked the arthritic index as compared to disease group from day 16 till the end of study. The maximum index was detected in DCG (4.17 ± 0.41) at day 28th that was restored significantly (*p* < 0.05–0.0001) in standard control (0.50 ± 0.54), CTAQ 600 (1.33 ± 0.51), CTAQ 300 (1.83 ± 0.41), CTAQ 150 (2.17 ± 0.75), CTME 600 (1.67 ± 0.52), CTME 300 (2.17 ± 0.41) and CTME 150 mg/kg (2.67 ± 0.52) treated groups in contrast to diseased rats as presented in Figure 4c.

### 3.7. Effect on Blood Parameters

**Figure 5:**
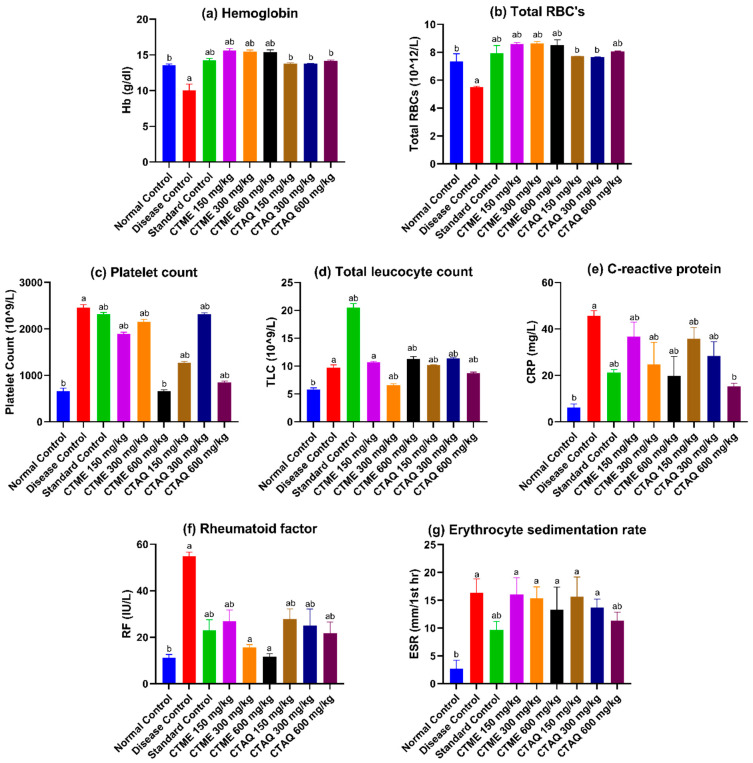
Influence of *C. tuberculata* extracts on hematological parameters. Data as mean ± S.D and considered significant as compared to a and b. Where a: NCG; b: DCG.

The CFA-induced arthritis in rats resulted in alteration of hematological and biochemical parameters. The Hb, RBC’s and total leucocyte counts (TLC) were notably (*p* < 0.0001) reduced in DCG in contrast to normal rats that notably (*p* < 0.05–0.0001) restored with CTME, CTAQ and methotrexate treated groups (Figure 5a,b,d). ESR was notably raised in DCG (16.33 ± 2.52 mm/h), in contrast to normal rats which saw significantly (*p* < 0.01) reduced values in the standard control (9.67 ± 1.53 mm/h) and CTAQ 600 (11.33 ± 1.53 mm/h)-treated arthritic rats as compared to DCG (Figure 5g).

The systemic biomarkers like CRP (45.6 ± 2.25 mg/L) and RF (54.93 ± 1.72 IU/L) were increased significantly in the disease group in contrast to the normal group as shown in Figure 5e,f. However, treatment with CTME, CTAQ and methotrexate (CRP: 21.27 ± 1.27 mg/L; RF: 23.03 ± 4.52 IU/L) profoundly (*p* < 0.05–0.0001) restored CRP and RF levels in treated arthritic rats, in contrast to DCG.

**Figure 6:**
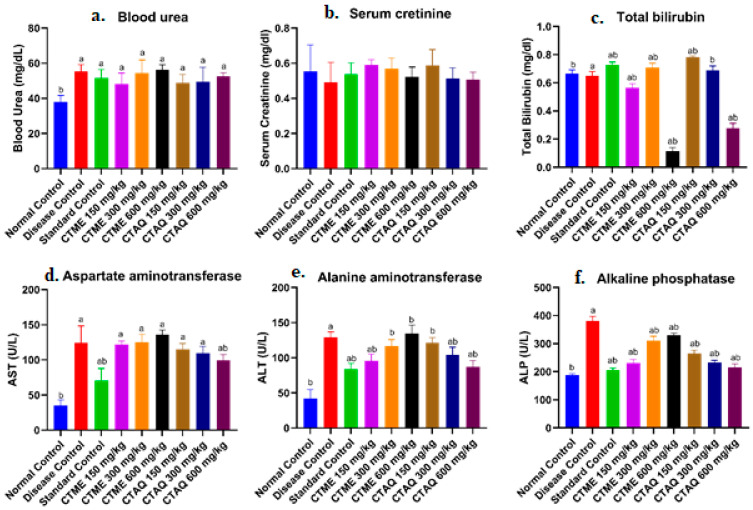
Effect of *C. tuberculata* extracts on kidney and liver function parameters. Values as mean ± S.D; (*n* = 6), Results were significant as equated to a and b. Where a: NCG; b: DCG.

The CFA-induced arthritis in rats resulted in an elevated levels of platelet counts, ALP, ALT and AST, as obvious in DCG in contrast to normal rats. Treatment with CTME, CTAQ and standard drugs caused a significant (*p* < 0.0001) reduction in platelet count and ALP values in comparison to DCG (Figure 6f). The ameliorating effect of CTME at all dosage levels and CTAQ at 150, 300 mg/kg on AST was insignificant (Figure 6d). The raised level of ALT in DCG was restored (*p* < 0.05) in methotrexate and CTME 150-, 300- and CTAQ 150–600- mg/kg treated groups (Figure 6e). The bilirubin level in all groups remained within normal range.

Treatment with CTME, CTAQ and methotrexate had an insignificant effect on the kidney function test (Figure 6a,b). However, the levels of these parameters lie between the normal ranges.

### 3.8. Effect on Histopathology of Arthritic Joints

**Figure 7:**
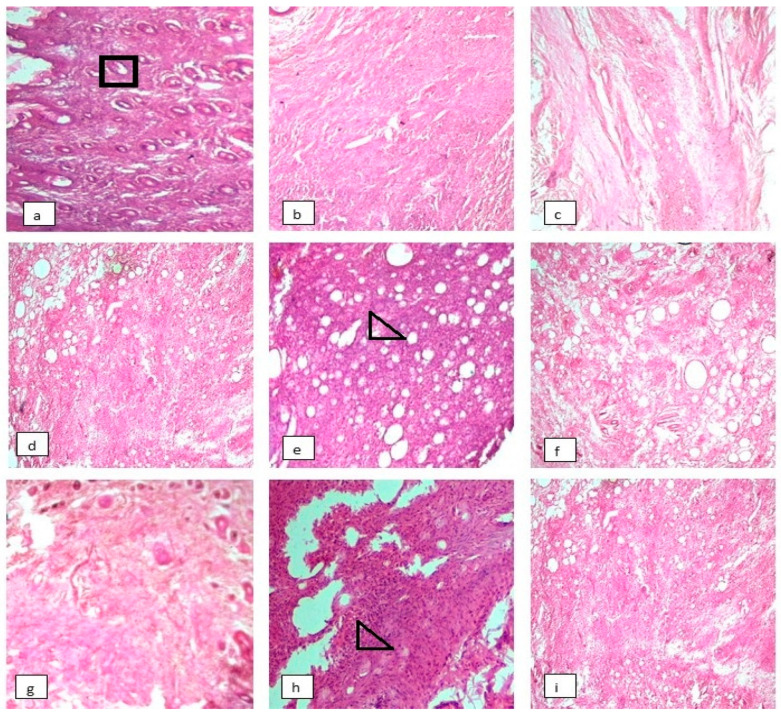
Effect of *Caralluma tuberculata* on histopathology of arthritic rats at 10× magnification. The tissues were stained with H & E stains. Here (**a**): Normal control group; (**b**): Disease control group; (**c**): Standard control group; (**d**): CTME150; (**e**): CTME 300; (**f**): CTME 600; (**g**): CTAQ 150; (**h**): CTAQ 300 and (**i**): CTAQ 600. Where square showed hair follicle and triangle showed chronic inflammation.

The ankle joint histopathology showed marked inflammation, infiltration of mononuclear cells, formation of pannus and bone erosion in the DCG while the synovial membrane was normal with no inflammation, bone or cartilage erosion in the normal control group (Figure 7a,b). Treatment with both plant extracts and the methotrexate-treated group showed reduction in inflammation, infiltration with mononuclear cell, bone erosion and pannus formation. Moreover, histopathological results revealed maximum healing in CTME and CTAQ 600 mg/kg treated groups as presented in Figure 7f,i.

### 3.9. Effect on Arthritic Indices

A pointedly high arthritic index was observed in DCG. The treatment groups showed mild and moderate inflammation, except for CTME 150 (2.6 ± 0.54) which showed severe inflammation as shown in Table 3. The minimal effect on pannus formation, inflammation and bone erosion was exhibited by CTAQ 600 mg/kg treated group. Both the plant extracts at dose of 600 mg/kg were insignificantly different as compared to standard control group.

### 3.10. Immune Organ Weight

**Figure 8:**
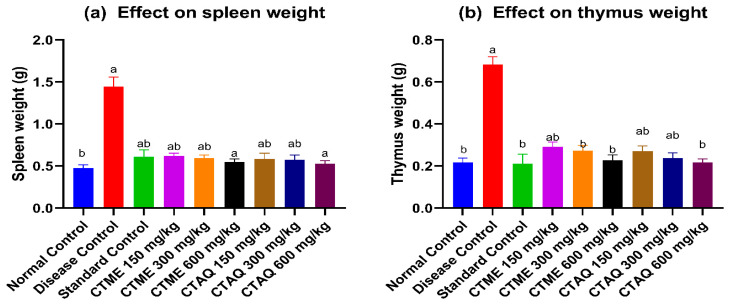
Effect of *Caralluma tuberculata* on immune organs weight. Values were expressed as mean ± S.D (*n* = 6). Results were significant (*p* < 0.05) as equated to a and b. Where a: NCG; b: DCG.

After 28 days of studying CFA polyarthritis, it was found that the spleen (1.44 ± 0.11 g) and thymus (0.68 ± 0.04 g) weight elevated in the diseased control animals in comparison to the normal rats. The weight of organs restored considerably (*p* < 0.05) in CTAQ, CTME at 600 mg/kg and standard therapy treated groups (spleen: 0.61 ± 0.08 g; thymus: 0.21 ± 0.05 g) as mentioned in Figure 8a,b.

### 3.11. Effect on Oxidative Stress

**Figure 9:**
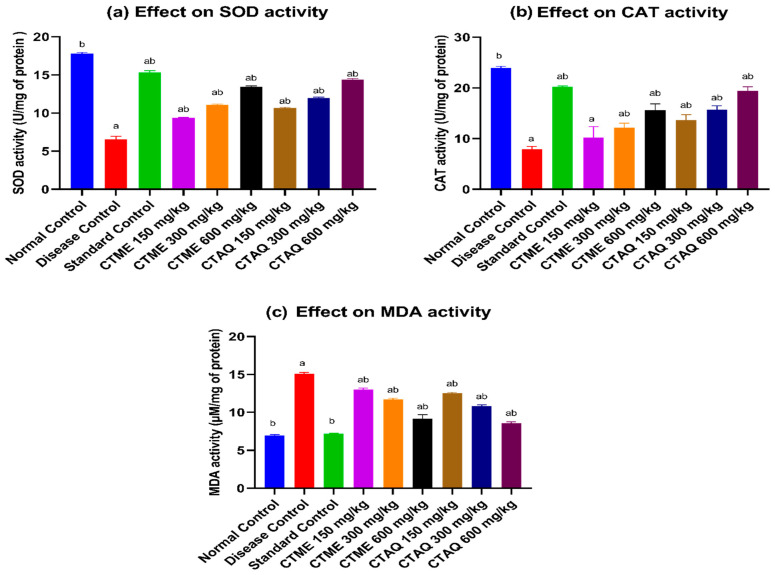
Effect of *C. tuberculata* on antioxidant level after CFA-induced arthritis. Values as mean ± S.D (*n* = 6). Results were significant (*p* < 0.001–0.0001) as equated to a and b. Where a: NCG; b: DCG.

It was noticed that there was a decreased activity (U/mg of protein) of SOD (6.56 ± 0.38) and CAT (8.25 ± 1.44) in 10% *w*/*v* liver homogenate of DCG as compared to the normal group (SOD: 17.77 ± 0.18 U/mg of protein; CAT: 24.49 ± 2.38) in the CFA-induced arthritic study. The treatment with both plant extracts and standard drugs significantly augmented the SOD and CAT activities in arthritic rats as shown in Figure 9a,b respectively, at all tested dosage levels that were notably varied from diseased control group. The CTME (SOD: 13.45 ± 0.10; CAT: 15.63 ± 1.22), CTAQ at 600 mg/kg (SOD: 14.37 ± 0.15; CAT: 19.42 ± 0.82) and methotrexate (SOD: 15.33 ± 0.21; CAT: 20.23 ± 1.32) expressively (*p* < 0.0001) reinstated the antioxidant enzyme activities as equated to DCG.

The concentration of MDA was profoundly upraised in the disease group (15.08 ± 0.19 µM/mg of protein) in comparison to the normal control (6.94 ± 0.14 µM/mg of protein). The treatment with CTME (9.15 ± 0.52 µM/mg of protein), CTAQ at 600 mg/kg (8.58 ± 0.18 µM/mg of protein) and standard control substantially (*p* < 0.0001) reinstated the MDA concentration in liver homogenate of treated arthritic rats as compared to diseased control rats (Figure 9c).

### 3.12. Effect on Gene Expression

**Figure 10:**
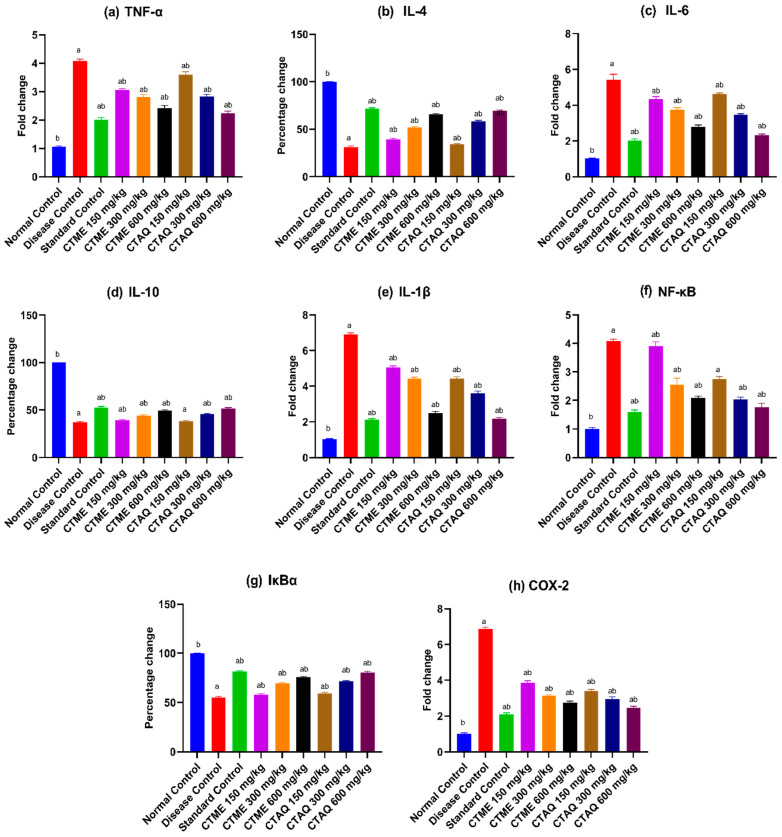
Effect of *C. tuberculata* extracts on gene expression in arthritic rats. Values as mean ± S.D (*n* = 6). Here ‘a’ and ‘b’ showed significantly different at *p* < 0.0001 in contrast to NCG and DCG respectively.

After 28 days of study, the mRNA expression of different inflammatory biomarkers was quantified by real-time PCR. A substantial (*p* < 0.0001) upregulation in the mRNA expression level of TNF-α, IL-6, -1β, and NF-κB was noticed in the diseased control group (TNF-α: 4.08 ± 0.06-fold, IL-6: 5.43 ± 0.31-fold, IL-1β: 6.9 ± 0.09-fold, NF-κB: 4.08 ± 0.07-fold) in contrast to normal group. The level of those pro-inflammatory biomarkers were notably (*p* < 0.0001) reinstated in standard control, CTAQ 600 and CTME 600 mg/kg treated groups in contrast to DCG as presented in Figure 10a,c,e,f.

The levels of COX-2 were increased noticeably (*p* < 0.0001) in diseased group (6.86 ± 0.11-fold) in contrast to normal while noticeably restored in standard group (2.09 ± 0.09-fold), CTAQ 600 (2.45 ± 0.09-fold) and CTME 600 mg/kg (2.75 ± 0.08-fold) treated animals in contrast to DCG (Figure 10h).

A substantial (*p* < 0.0001) downregulation in IL-4,-10, and I-κBα expression in the diseased control group (IL-4: 31.27 ± 1.12%, IL-10: 37.09 ± 0.9%, I-κBα: 55.24 ± 1.04%) in comparison to normal group in CFA induced polyarthritis. While a substantial (*p* < 0.0001) upregulation in the expression of these anti-inflammatory biomarkers in the standard control group (IL-4: 71.49 ± 1.19%, IL-10: 52.3 ± 1.35%, I-κBα: 81.31 ± 0.77%), CTAQ 600 (IL-4: 69.48 ± 0.85%, IL-10: 51.61 ± 0.9%, I-κB: 80.44 ± 1.14%) and CTME 600 mg/kg (IL-4: 65.86 ± 0.85%, IL-10: 49.13 ± 1.06%, I- κBα: 75.66 ± 0.77%)-treated groups was observed in contrast to diseased control animals as mentioned in Figure 10b,d,g.

## 4. Discussion

The RA is an autoimmune syndrome characterized by chronic inflammation associated with pain, stiffness and the destruction of synovial joints [32]. This research was designed to evaluate the anti-arthritic potential of *C. tuberculate* extracts using various in vitro and in vivo tests.

The HPLC analysis identified different phenolic and flavonoid contents like catechin, ferulic acid, quercetin, gallic acid, caffeic acid and vanilic acid. It was previously reported that the presence of compounds like ferulic acid, quercetin, gallic acid and vanilic acid was responsible for antioxidant and anti-inflammatory potential [33]. The presence of these compounds in CT extract had also previously been reported [34]. In RA, denaturation of tissue proteins occurs, causing production of auto-antigens that results in the destruction of synovial membrane and cartilage. Both plant extracts showed significant reduction in albumin denaturation, dose-dependently, and was similar to that of NSAIDs having known anti-inflammatory potential against arthritis. The protection from protein denaturation might be due to the presence of fatty acids, phytosterols, phenols, flavonoids, alkaloids and tannins in the plant extracts as reported in previous studies [35].

Lysosomal components are produced from neutrophils and macrophages during inflammation, to kill surrounding cells and tissues. Anti-inflammatory medicines inhibit inflammation by stabilizing the membrane or by inhibiting lysosomal enzymes [12]. The HRBC assay was performed to evaluate the membrane stabilizing potential of CTME and CTAQ against membrane rupturing factors like heat and hypotonicity. Both extracts of CT exhibited notable membrane stabilization dose-dependently however, maximum stabilization was exhibited by CTME 1600 µg/mL.

The phytochemicals like quercetin and gallic acid are thought to prevent lysosomal degradation due to their free radical-scavenging activity [9]. Therefore, it is assumed that the presence of phenols and flavonoids in CTME and CTAQ are responsible for the protection and stabilization of the membrane. The membrane stabilization assay results revealed that the plant extracts of CT exert a similar dose-dependent effect to that of piroxicam.

Weight loss is associated with the progression of RA. An increase in body weight along with a decrease in paw swelling indicates an improvement of RA [36]. The study revealed that both plant extracts significantly reduced paw swelling and restored body weight at the end of study in comparison to the arthritic control. The immune organ weights were also restored in extracts treated groups in contrast to the arthritic control. One of the major clinical features of RA is anemia i.e., decreased levels of Hb and RBCs while platelet count is increased in RA [37]. The hematological testing revealed that both plant extracts significantly restored Hb, RBCs, platelet count and TLC in treated rats, as compared to disease group, which was evidenced from the improvement in arthritic rats.

Liver impairment is also associated with arthritis and an increase in ALP levels is an indication of bone erosion and osteoporosis. By measuring enzyme activity in the serum, tissue damage was estimated [21]. The damaged organs release AST from its cells that results in an elevated level of AST as reported in a current study into DCG. A significant fall in the levels of ALP, ALT and AST was reported with treatment groups in contrast to the diseased control rats in this study. This reduction of LFTs indicates the anti-inflammatory potential of both extracts [38].

Physical examination of disease progression is estimated by measuring arthritic score; this was notably reduced with treatment in contrast to DCG throughout the study period. The estimation of disease stage is done by measuring erythrocyte sedimentation rate (ESR), C-reactive protein (CRP) and RF value in serum. These are also biomarkers for the estimation of systemic inflammation [10,39]. Increased levels of these parameters indicate the progression of disease, as observed in diseased control rats in the current study. In inflammation, a high amount of fibrinogen is produced in blood that results in the sticking of RBCs to each other. ESR, an inflammatory biomarker, is the measure of the rate at which RBC’s sediments during a period of 1 h. ESR was significantly (*p* < 0.01) reduced in standard control group and CTAQ 600 mg/kg group as compared to DCG. The CRP production is aggravated by an increased level of pro-inflammatory cytokines such as IL-6 and TNF-α [40]. The study showed the levels of these biomarkers were restored profoundly with CTME, CTAQ and methotrexate in comparison to the DCG.

During an inflammatory process, pro-inflammatory cytokines (TNF-α, IL-1β and IL-6) are released from activated monocytes and macrophages. TNF-α activates the release of two other pro-inflammatory cytokines that results in the release and infiltration of leukocytes resulting in vasodilation at the site of edema. TNF-α and IL-1β stimulate the production of MMPs inside the inflamed joint that exacerbated joint destruction. In order to cope with this, inhibition of pro-inflammatory cytokines is mandatory [41] to halt disease progression as well. The plant extracts and methotrexate showed significant (*p* < 0.05–0.0001) downregulation of mRNA expression of pro-inflammatory cytokines and COX-2 in treated rats, while notably upregulating the expression of anti-inflammatory cytokines (IL-4 and IL-10) as compared to DCG. This showed the symptomatic and systemic relief by CT extract in CFA-induced polyarthritis as evident from histology slides of ankle joints of treated rats. The similar findings on arthritic parameters were exhibited in various previous studies [42,43].

The NF-κB is a member of group of transcription factors and responsible for different gene expressions that are involved in immunity and various inflammatory processes. The NF-κB have strong effect on synovial tissues in RA and is detected in activated form in human synovial tissues at early and later stages of inflammation [44]. The I-κBα acts as an auto-regulatory feedback loop to suppress NF-κB activity and keeps it in an inactive form in the cytosol [45]. Upon stimulation, I-κB kinase (IKK) phosphorylates I-κB resulted in the translocation of NF-κB which then directs synthesis of various inflammatory markers. IKK is activated via various stimuli that includes cytokines, mitogens, microbial components, growth factors, and stress agents [46]. It is reported in previous studies that the presence of quercetin and catechin in plant extracts favors anti-arthritic and antioxidant potential [42,43,47]. In the current study, both extracts notably modulated oxidative stress biomarkers in liver tissue homogenate and I-κB and NF- κB in treated rats that were not reported earlier. CTAQ is more active than CTME in mitigating arthritic symptoms. The acute and subacute toxicity of QL aqueous extract exhibited no mortality, hema-, hepato- and nephrotoxicity in our other study (unpublished).

The 2-Methoxy-4-vinylphenol exhibited antimicrobial, antioxidant, anti-inflammatory, analgesic [48]. The γ-tocopherol and vitamin E are strong antioxidant, anti-inflammatory and anticancer agents and aid the nerves and muscles of the body to work properly. Vitamin E exhibit anti-inflammatory action via reducing CRP and proinflammatory cytokines [49].

Phytosterols such as stigmasterol, β-sitosterol, campesterol exhibited antioxidant, anti-inflammatory and antimicrobial activity. These exhibited anti-inflammatory potential via reducing COX-2, nitric oxide, and proinflammatory cytokines [50,51,52]. Various fatty acids (n-Hexadecanoic acid, Heptadecanoic acid, 10,13-Octadecadienoic acid, 9,12-Octadecadienoic acid, 9,17-Octadecadienal, Octadecanoic acid) present in food exhibitedd antioxidant and anti-inflammatory action by inhibiting both cyclooxygenases [53]. 5-hydroxymethyfurfural exhibited anti-inflammatory and antioxidant activities [54]. Phytol exhibited anti-inflammatory, antioxidant, anticancer, antimicrobial and antidiabetic activities [55]. Lanosterol exhibited antifungal, anti-inflammatory and antioxidant actions [18]. Lupeol exhibited anti-inflammatory, wound-healing, antioxidant, and anticancer activities [19]. Thus all the compounds detected in HPLC and GC-MS analysis might be involved in the anti-arthritic action of *C. tuberculata*.

## 5. Conclusions

It is concluded from the findings that *C. tuberculata* extracts exhibited anti-arthritic effects via stabilizing membrane, inhibiting protein denaturation, reducing edema, and restoring body and immune organ weights. Moreover, these treatments restored altered blood parameters in treated rats. Both extracts exhibited downregulation of TNF α, IL-6, -1β, NF-κB, and COX-2, while notably upregulating IL-4, -10, and I-κB as compared to DCG. Additionally, both extracts noticeably (*p* < 0.001–0.0001) restored the SOD, CAT activities and MDA level in treated rats in contrast to DCG. The CTAQ exerted profound effects in masking disease symptoms as compared to CTME at 600 mg/kg. There is an immense need for the isolation of required phytochemicals from CTME and CTAQ that are responsible for the anti-arthritic activity. Other anti-inflammatory mechanisms of CT should be found out. Clinical trials should be conducted on this substance prior to its use as a nutraceutical.

## Figures and Tables

**Table 1:** HPLC analysis of *Caralluma tuberculata* extract.

CTME	**Compound**	**Peak No.**	**Retention Time (min)**	**Area** **(mV.s)**	**Height**	**Concentration (ppm)**
	Catechin	4	3.215	542,990.4	49,453.1	203.62
	Gallic acid	5	3.375	151,085.00	23,349.5	11.94
	Caffeic acid	7	7.694	1,330,149.4	165,364.3	43.23
	Ferulic acid	12	12.944	111,935.6	10,471.5	5.54
	Quercetin	17	24.735	583,585.5	46,390.1	55.44

**Table 2:** GC-MS analysis of *C. tuberculata* extract.

Peak No.	Peak Time (min)	Chemical Compound	Peak Area	Peak Area (%)
1.	5.010	Butanenitrile, 2,3-dioxo-, dioxime, O,O’-diacetyl-	1,481,878	0.33
2.	5.234	l-Guanidinosuccinimide	2,666,278	0.59
3.	5.400	3,4-Dihydroxybenzyl alcohol,tris(trimethylsilyl)-	1,711,708	0.38
4.	6.122	N-Isobutyl-sec-butylamine	4,454,248	0.98
5.	7.149	5-Hydroxymethylfurfural	3,075,712	0.68
5.	9.091	2-Methoxy-4-vinylphenol	2,013,622	0.44
7.	9.856	Ethane, 1,2-diethoxy-	2,617,942	0.57
8.	14.129	4-Methyl-2,5-dimethoxybenzaldehyde	12,671,034	2.78
9.	17.504	Tetradecanoic acid	2,845,228	0.62
10.	18.542	2-Propenoic acid, 2-methyl-, 1-methylethyl ester	1,936,230	0.43
11.	18.900	Dimethylphosphinacrylonitryl	2,395,846	0.53
12.	19.360	Neophytadiene	2,145,525	0.47
13.	19.852	D-Carvone	2,247,263	0.49
14.	20.125	Hexadecanoic acid, methyl ester	5,070,649	1.11
15.	20.248	1-Propoxypropan-2-yl nonanoate	20,180,612	4.43
16.	20.377	3-Ethyl-4-methyl-3-heptanol	1,495,429	0.33
17.	20.837	n-Hexadecanoic acid	30,618,380	6.72
18.	22.259	Heptadecanoic acid	2,723,170	0.60
19.	22.693	10,13-Octadecadienoic acid, methyl ester	4,406,647	0.97
20.	22.784	9,12,15-Octadecatrienoic acid, methyl ester	5,144,038	1.13
21.	22.949	Phytol	15,955,953	3.50
22.	23.404	9,12-Octadecadienoic acid (Z,Z)-	17,972,849	3.95
23.	23.490	9,17-Octadecadienal, (Z)-	16,459,178	3.61
24.	23.800	Octadecanoic acid	3,675,449	0.81
25.	24.003	3-Buten-2-ol, 2,3-dimethyl-	6,599,384	1.45
26.	24.287	Ethyl ether	5,283,067	1.16
27.	28.571	Cyclohexadecane, 1,2-diethyl-	1,575,111	0.35
28.	28.913	Glycerol 1-palmitate	10,427,195	2.29
29.	32.170	1,3,11-Dodecatriene	6,174,131	1.36
30.	32.802	Myristoyl chloride	1,730,016	0.38
31.	32.903	3-Buten-2-ol, 2,3-dimethyl-	3,430,046	0.75
32.	33.625	Silane, diethyldimethyl-	11,604,735	2.55
33.	33.957	Pentanoic acid, 4-methyl-, methyl ester	4,240,177	0.93
34.	35.086	Isopropyl myristate	3,430,717	0.75
35.	37.947	(Z)-epi-.beta.-Santalol	2,100,345	0.46
36.	39.776	γ -Tocopherol	20,513,277	4.50
37.	40.466	2-Amino-7-chloro-[1,3]thiazino [5,6-c]quinolin-4-one	3,022,677	0.66
38.	40.787	Phenol, 2,6-di-t-butyl-4-methyl-3-nitro-, acetate(ester)	1,899,459	0.42
39.	42.146	Vitamin E	24,222,842	5.32
40.	42.889	Olean-12-en-3-ol, acetate, (3.beta.)-	1,534,963	0.34
41.	44.194	Campesterol	32,976,594	7.24
42.	44.355	Silane, dimethylnonyloxypropyloxy-	5,280,442	1.16
43.	44.836	Stigmasterol	23,213,212	5.10
44.	46.484	β-Sitosterol	47,356,836	10.40
45.	47.099	β-Amyrin	5,507,688	1.21
46.	47.922	Androst-5-en-17-ol, 4,4-dimethyl-	11,314,420	2.48
47.	48.356	Lupeol	18,854,916	4.14
48.	49.618	Lanosterol	12,838,547	2.82

Activity against protein denaturation.

**Table 3:** Effect of *C. tuberculata* extracts on the arthritic index.

Arthritic Index	Disease Control	Standard Control	CTME (mg/kg)			CTAQ (mg/kg)		
			150	300	600	150	300	600
Pannus formation	2.6 ± 0.54	0.8 ± 0.84 ***	2.4 ± 0.55 ^ns^	2.2 ± 0.45 ^ns^	1.2 ± 0.84 **	1.8 ± 0.83 ^ns^	1.0 ± 0.71 **	0.6 ± 0.89 ***
Inflammation	2.8 ± 0.44	1.2 ± 0.83 ***	2.6 ± 0.54 ^ns^	1.8 ± 0.44 *	1.6 ± 0.55 **	1.6 ± 0.55 **	1.4 ± 0.55 **	1.0 ± 0.71 ****
Bone erosion	2.6 ± 0.55	0.4 ± 0.55 ****	1.6 ± 1.14 ^ns^	1.2 ± 0.84 **	0.8 ± 0.45 ***	1.2 ± 0.84 **	0.6 ± 0.54 ***	0.4 ± 0.55 ****

Results as mean ± S.D. *, **, ***, **** *p* < 0.05, 0.01, 0.001, 0.0001 as equated to DCG; ns: Non-significant.

## Data Availability

Authors declare that all the data supporting the findings of this study are included in the article.

## Sample Availability

Samples of the compounds are not available from the authors.
